# Dynamic evolution of *Panax* species

**DOI:** 10.1007/s13258-021-01047-6

**Published:** 2021-02-20

**Authors:** Hyeonah Shim, Nomar Espinosa Waminal, Hyun Hee Kim, Tae-Jin Yang

**Affiliations:** 1 Department of Agriculture, Forestry and Bioresources, Plant Genomics and Breeding Institute, Research Institute of Agriculture and Life Sciences, College of Agriculture and Life Sciences, Seoul National University, 1 Gwanak-ro, Gwanak-gu, Seoul, 08826 Korea; 2 Department of Chemistry and Life Science, Bioscience Institute, Sahmyook University, Seoul, 01795 Republic of Korea

**Keywords:** *Panax ginseng*, Evolution, Whole genome sequence, Whole genome duplication

## Abstract

**Background:**

*Panax ginseng* is one of the most valuable medicinal plants in Korea. However, deciphering its full genome sequence information for crop improvement has been hampered due to its complex genomic, genetic, and growth characteristics. Many efforts have been made in the past decade to overcome these limitations and understand the genome structure and the evolutionary history of *P. ginseng*.

**Methods:**

This review aims to discuss the current status of genomic studies on *P. ginseng* and related species, and the experimental clues suggesting phylogenetic classification and evolutionary history of the genus *Panax*.

**Conclusion:**

The development of sequencing technologies made genome sequencing of the large *P. ginseng* genome possible, providing fundamental information to deciphering the evolutionary history of *P. ginseng* and related species. *P. ginseng* went through two rounds of whole genome duplication events after diverging from the closest family Apiaceae, which was unveiled from complete whole genome sequences. Further in-depth comparative genome analysis with other related species and genera will uncover the evolutionary history as well as important morphological and ecological characteristics of *Panax* species.

## Introduction

*Panax ginseng* (named Korean ginseng or Asian ginseng) is one of the most valuable representative medicinal plants in Korea, which is also known worldwide. Ginseng, along with other *Panax* species distributed in Asia and North America, belongs to the genus *Panax* of the Araliaceae family, which contains around 1,500 species (Plunkett et al. 1996; Wen et al. 2001). Ginseng also naturally grows in parts of China and Russia (Grushwitsky 1961; Wen and Zimmer 1996; Zhuravlev et al. 2008). It has 2*n* = 4*x* = 48 chromosomes (Waminal et al. 2012) with an allotetraploid genome of ~ 3.6 Gbp (Kim et al. 2018). Ginseng genetic studies, and breeding in general, are quite challenging due to its limited growth characteristics such as a long generation time of four years and only a few seeds (~ 40) set per plant starting from the fourth year (Choi et al. 1998; Choi 2008; Jayakodi et al. 2014).

*The Panax* genus comprises about 17 species (Zhang et al. 2020). *Panax quinquefolius* L., also known as North American ginseng (Kim et al. 2016b), grows in North America along with *Panax trifolius* (Wen and Zimmer 1996). *Panax notoginseng* Burkill grows and is cultivated in China (Schorger 1969; Wen and Zimmer 1996), while *Panax vietnamensis* Ha et Grushv exists in certain regions of Vietnam (Van Duy et al. 2016). These *Panax* species are cultivated in their respective countries for their high medicinal value and scarcity.

Here, we review the genomics research in ginseng and the related species, with special emphasis on the whole genome assembly status, genomic characteristics in *Panax* species as well as the evolution of *Panax* species as revealed by comparative genomics analyses. We also present the on-going genomics studies in related species outside the genus *Panax* but within the Araliaceae family.

## Status of genomic research in the genus *Panax*

Currently, whole genome sequence information is available only for two species, the allotetraploid *P. ginseng* (Kim et al. 2018) and the diploid *P. notoginseng* (Chen et al. 2017; Zhang et al. 2017). Xu et al. (2017) assembled the whole *P. ginseng* genome and obtained a 3.41 Gbp draft assembly with a scaffold N50 of 108.71 kb and annotated 42,006 genes. In 2018, a draft sequence of the major *P. ginseng* cultivar of Korea, ‘Chunpoong’, was completed and published (Kim et al. 2018). This draft sequence consisted of 2.98 Gbp with a scaffold N50 of 569.02 kb and 59,352 annotated genes. Sequence and annotation information as well as basic genomic analysis tools provide a golden standard via an open-access platform database for researchers around the world to utilize for ginseng research (Jayakodi et al. 2018).

Other genomic studies on the whole genome sequence are available for *P. notoginseng*. In 2017, Zhang et al. completed a 1.85 Gb whole genome with a scaffold N50 of 157.81 kb and annotated 34,369 genes (Zhang et al. 2017). In the same year, Chen et al. reported a 2.39 Gb whole genome sequence with a scaffold N50 of 96 kb, annotating 36,790 genes (Chen et al. 2017). In 2020, Fan et al. completed a 2.25 Gb assembly consisting of 16,469 scaffolds with a contig N50 220.89 kb and annotated 39,452 genes (Fan et al. 2020). Jiang et al. reported a significantly improved chromosome-level assembly of *P. notoginseng*. The 2.66 Gb reference genome has a scaffold N50 of 216.47 Mb with 37,606 genes annotated (Jiang et al. 2020). The assembly statistics of the *P. ginseng* and *P. notoginseng* genome are provided in Table 1.

**Table 1:** Whole genome sequences available for *Panax* species

	*Panax ginseng*		*Panax notoginseng*			
	Xu et al. (2017)	Kim et al. (2018)	Chen et al. (2017)	Zhang et al. (2017)	Fan et al. (2020)	Jiang et al. (2020)
No. of scaffolds	337,439	9845	122,131	76,517	16,469	219
Total length	3.41 Gb	2.98 Gb	2.39 Gb	1.85 Gb	2.25 Gb	2.66 Gb
Contig N50	22.00 kb	22.54 kb	16 kb	13.16 kb	220.89 kb	1.12 Mb
Scaffold N50	108.71 kb	569.02 kb	96 kb	157.81 kb	–	216.47 Mb
Longest scaffold	1.30 Mb	3.64 Mb	834.33 kb	1.19 Mb	7.10 Mb	295.55 Mb
No. of genes	42,006	59,352	36,790	34,369	39,452	37,606

## Molecular cytogenetic tools in ginseng genome analysis

**Fig. 1:**
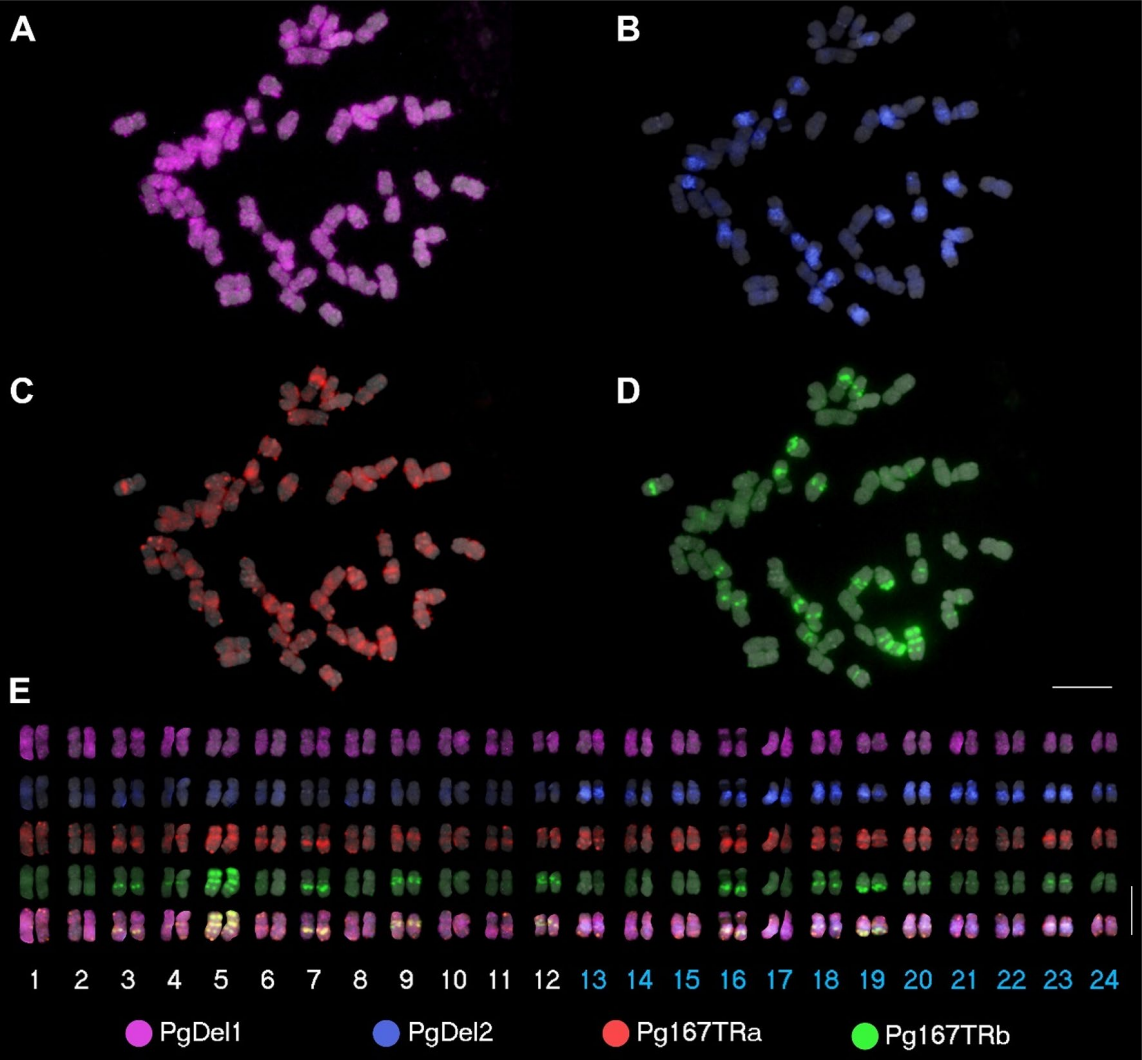
Simultaneous detection of repeat elements in *P. ginseng* chromosomes using multi-color FISH. FISH signals are shown for transposable element (**a,** **b**), and satellite DNA (**c,** **d**) probes. **a** *PgDel1*-Cy5, **b** *PgDel2*-DEAC, **c** Pg167TRa-Texas Red, and **d** Pg167TRb-Alexa Fluor 488. **e** Chromosomes from **a**–**d** were arranged according to *PgDel2* signal and chromosome length. The last row in **e** show the merged signals of the four repeats. Bars = 10 µm (color figure online)

Genomic features can be analyzed through molecular cytogenetics in which genome size, chromosome number, and clues to understand genome evolution can be obtained. With the development of cytogenetic tools such as fluorescence in situ hybridization (FISH) (Pinkel et al. 1986), it became much easier to analyze the karyotype of plant species and utilize them for taxonomic studies. The first *P. ginseng* karyotype was reported by Waminal et al. (2012), which was later improved by the identification of abundant satellite DNA, Pg167TR (Choi et al. 2014; Waminal et al. 2017). Identification of additional repeat elements widened the scope of cytogenetic markers for ginseng genome analysis (Fig. 1).

Advances in cytogenetics also made possible the sequencing and analysis of individual chromosome through flow sorting of chromosomes (Doležel et al. 2014). Some example applications of sequencing of flow-sorted chromosomes include cereal crops such as *Triticum aestivum* (IWGSC 2014), *T. dicoccoides* (Akpinar et al. 2018), and *Hordeum vulgare* L. (Lysák et al. 1999). Coupling flow sorting with sequencing techniques provides a great platform for high-quality genome sequencing and analysis for plants, particularly those with large genomes because many plant species usually have complex genome structures, large genome sizes, and high repetitive content as well as high levels of heterozygosity.

## Two whole genome duplication events in *Panax ginseng*

Structure and characteristics of ginseng genome were elucidated by the draft genome sequence of *P. ginseng* (Kim et al. 2018). Although the available genome assemblies are not in chromosome or pseudochromosome levels, the scaffold information holds critical information and clues on duplication events throughout the evolutionary history of *P. ginseng*. Whole genome assembly resulted in highly homologous sequences between scaffolds and paralogous genes with similarities up to 99%. Reference-guided super-scaffolding, using the *Daucus carota* genome (2*n* = 2*x* = 18) as a reference, indirectly suggested that *P. ginseng* went through two rounds of genome duplications independent from those that occurred in the carrot genome. Moreover, cytogenetic analysis of the genic regions of scaffolds showed that the probe signals appeared in the same chromosome as well as different chromosomes. These genomic characteristics derived from sequence and chromosome analyses of *P. ginseng* suggested a duplication event.

**Fig. 2:**
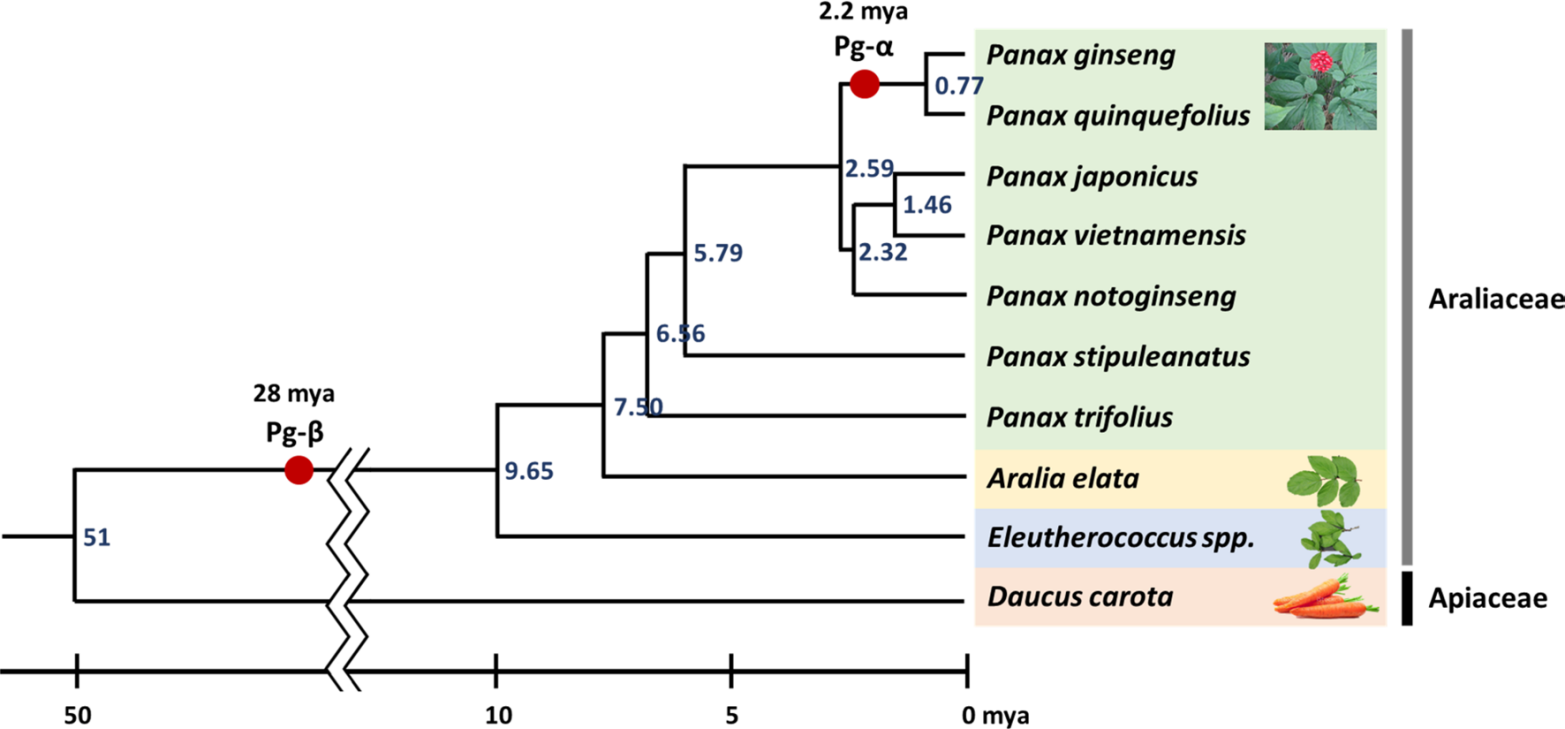
Two whole genome duplication events occurring for *Panax* species. Reorganized and redrawn based on divergence time estimations of Lee et al. (2017), Kim et al. (2017), and Kim et al. (2018)

Duplication events were confirmed by synonymous substitution (Ks) values calculated between orthologous gene clusters collected from *P. ginseng* and four dicots that include *Arabidopsis thaliana*, *Vitis vinifera*, *Solanum lycopersicum*, and *Daucus carota*. Ks values suggested a divergence event between Araliaceae and Apiaceae around 51 million years ago (mya). Moreover, whole genome duplication events of *P. ginseng*—an ancient genome duplication around 28 mya common to all Araliaceae species, and a relatively recent event around 2.2 mya unique to *Panax* species—have been inferred from Ks calculation of paralogous gene pairs in *P. ginseng* (Fig. 2) (Kim et al. 2018). Divergence time estimation results were consistent with previous estimations using expressed sequence tags (Choi et al. 2013), repeat composition (Choi et al. 2014), and complete chloroplast genome and nrDNA sequences (Kim et al. 2017).

## Repeat explosion contributed to the speciation, adaptation, and genome size expansion in *Panax*

**Fig. 3:**
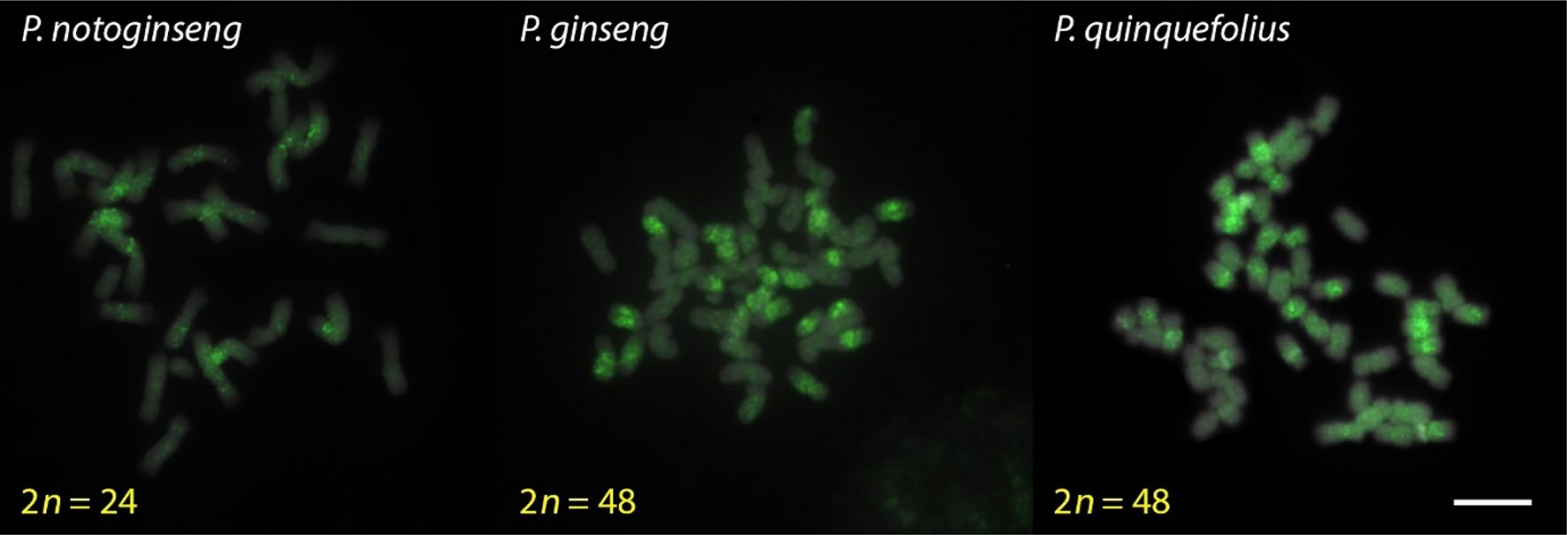
Distribution of *PgDel2* retrotransposon in diploid *P. notoginseng* and tetraploid *P. ginseng* and *P. quinquefolius*. In *P. notoginseng*, *PgDel2* was dispersed in pericentromeric regions of all chromosomes. In tetraploid *Panax*, *PgDel2* was concentrated at pericentromeric regions in 24 out of 48 chromosomes. Bar = 10 µm

Major repeat components were first identified by sequencing the bacterial artificial chromosome (BAC) sequences of ginseng (Choi et al. 2014; Jang et al. 2017). Analyses showed that most of the BAC sequences were comprised of repeats, and the two main repeat types were Ty3/Gypsy, consisting of *PgDel*, *PgTat*, *PgAthila* (Choi et al. 2014; Jang et al. 2017), and Ty1/Copia which contains *PgTork* and *PgOryco* (Choi et al. 2014). The repeat proportion within the genome could be calculated by mapping whole genome shotgun sequence reads, which showed that *PgDel* is the most abundant, *PgDel1* sub-family being the most abundant (about 25%) in *P. ginseng* (Lee et al. 2017). The distribution has been visualized by FISH (Fig. 1) (Choi et al. 2014; Lee et al. 2017). Moreover, FISH analysis showed that *PgDel1* is distributed across all 24 chromosome pairs while *PgDel2* occupies only half of the chromosome complement; it was detected in only 12 chromosome pairs (Fig. 3) (Choi et al. 2014). This acts as strong evidence of the recent allotetraploidization event for *P. ginseng* emergence (Choi et al. 2014).

The major repeats found in *P. ginseng* were also calculated in other *Panax* species. Five *Panax* species and *Aralia elata* were analyzed for their repeat content, and *PgDel1* showed the most dynamic proportion in the six species analyzed. *PgDel1* was even more abundant in *P. quinquefolius*, covering almost 35% of the genome. Considering the size of *PgDel1* being around 10 kb, the increase in *PgDel1* in *P. quinquefolius* accounts for almost 1 Gbp of genome expansion. Compared to *Panax* species, *A. elata* had small amounts of *PgDel1*, but was more abundant in *PgTork* (Lee et al. 2017). Major retrotransposons contributed to the speciation of the *Panax* species, which can be most evidently seen in the case of *P. ginseng* and *P. quinquefolius*. Although *P. quinquefolius* (~ 4.9 Gb) diverged from *P. ginseng* (~ 3.6 Gb) less than 1 mya, the genome size differs by 1.3 Gb which is most likely caused by the expansion of *PgDel1* (Lee et al. 2017).

## Phylogenetic relationship among *Panax* genus and related species using sequence information

*P. ginseng* and *P. quinquefolius* are allotetraploid species (2*n* = 2*x* = 48) that grow in freezing winter regions while *P. notoginseng*, *P. japonicus*, *P. vietnamensis*, and *P. stipuleanatus* are diploid species inhabiting Asian countries in high altitude non-freezing regions. Meanwhile, *P. trifolius* is a diploid species that exist in the cold regions of North America. Complete chloroplast genome sequences and 45S nrDNA of target species as well as *Panax* related species were obtained by using a de novo assembly method using low-coverage whole-genome sequence (dnaLCW) (Kim et al. 2015) and compared for genetic diversity and clues for evolution (Kim et al. 2017). Chloroplast genome and 45S nrDNA sequences were compared to decipher the phylogeny of *Panax* species and related species and their classification. After diverging from the Apiaceae family 51 mya, *Eleutherococcus* species diverged from *Panax* and related genera around 10 mya. Then, *Aralia elata*, the closest relative to *Panax* species, diverged around 8 mya. Finally, the tetraploid *P. ginseng* and *P. quinquefolius* diverged around 1 mya after the second whole genome duplication event within the *Panax* genus at 2.2 mya (Fig. 2) (Kim et al. 2017). These results were generally in concordance with those of other reports (Lee et al. 2017; Kim et al. 2018). With the variation information derived from polymorphisms within the chloroplast genome sequence, molecular markers were designed and applied to distinguish the different species for authentication purposes which can be applied to various bioproducts (Nguyen et al. 2017, 2020).

## Migration and adaptation created the current genetic pool of *Panax* species

**Fig. 4:**
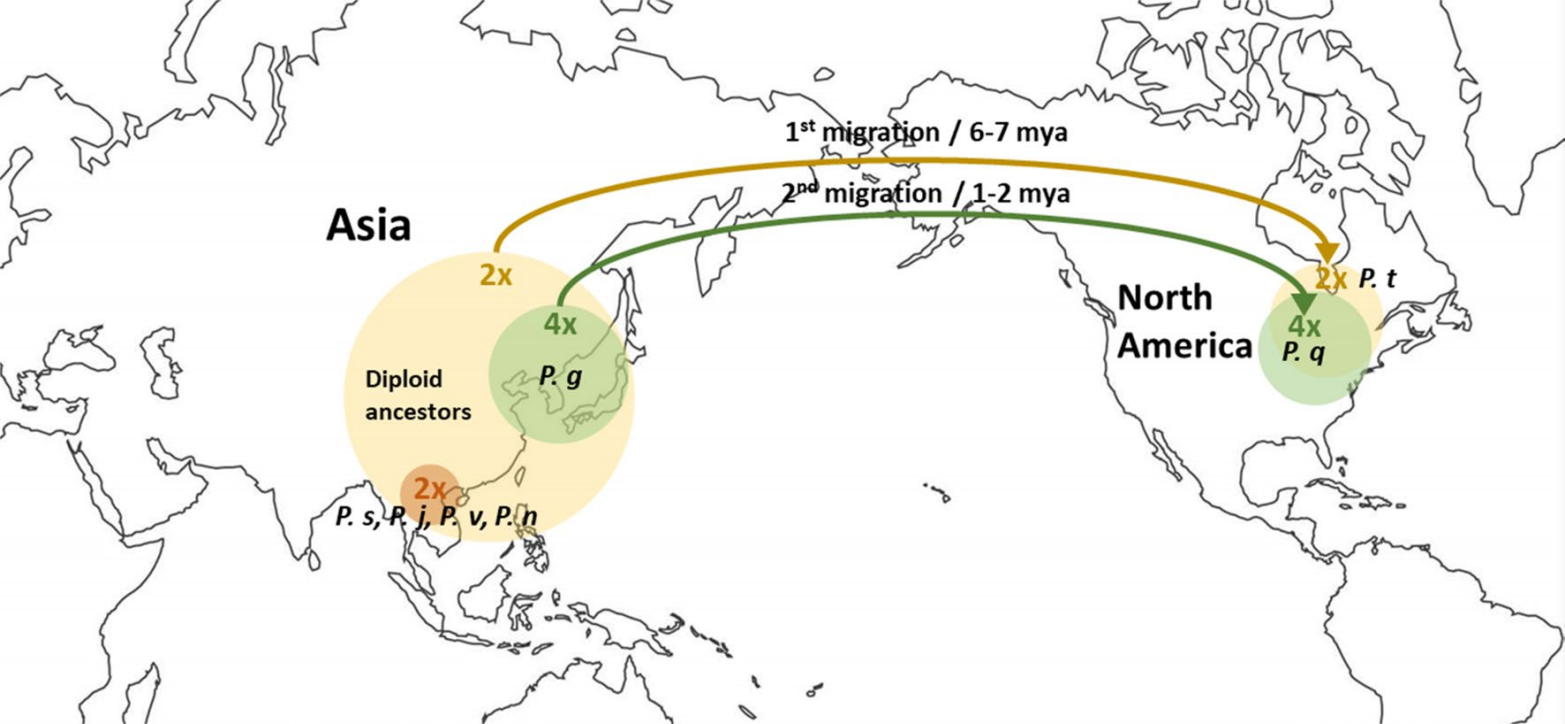
Two intercontinental migration of *Panax* species recreated based on Kim et al. (2018). *P. g* *P. ginseng*, *P. s* *P. stipuleanatus*, *P. j* *P. japonicas*, *P. v* *P. vietnamensis*, *P. n* *P. notoginseng*, *P. t* *P. trifolius*, *P. q* *P. quinquefolius*

Kim et al. (2018) suggests the current distribution of *Panax* species based on two intercontinental species migrations and cycles of ice ages and global warming. Ancestor diploid species of *Panax* could have started off inhabiting larger regions of Asia than those inhabited today. One diploid species, *P. trifolius* made the first intercontinental migration to the North American continent 6–7 mya. Then, glaciation caused the diploid species residing in Asia to face extinction because they do not have overwintering abilities. Two likely extinct ancestral diploid species created an allotetraploid ancestor of *P. ginseng* around 2 mya which may have survived in Northeast Asia while gaining overwintering properties. Meanwhile, most diploids could not survive in Northern Asia or lower altitudes in Southern Asia due to global warming, and the remaining moved up to higher altitudes in Southern Asia. While a tetraploid *P. ginseng* ancestor occupied the Northeast Asian regions, a second intercontinental migration occurred around 1 mya which made a possible ancestor of *P. quinquefolius* to North America during glacial migration (Fig. 4).

## Further studies

Studies related to ginseng and related genera are ongoing. Although a *P. ginseng* reference genome is now available in literature (Jayakodi et al. 2018; Kim et al. 2018), this is still in a draft sequence level which needs considerable improvement to achieve chromosome-level assembly to be more reliable for sophisticated downstream analyses. Recent sequencing technologies such as Oxford Nanopore Sequencing Technologies and Hi-C, among others, have been recently adapted to improve the present genome assembly. These technologies have significantly refined the *P. ginseng* assembly to a pseudochromosome level, with super-scaffolds roughly representing the 24 chromosomes of *P. ginseng* that are being confirmed with oligo-FISH and genotyping by sequencing (GBS) methods (unpublished data).

Moreover, the evolution of the Araliaceae family is interesting to note because the *Panax* genus and the related genera are morphologically and ecologically diverse. Cytogenetic studies of 15 Araliaceae species revealed that genome sizes and chromosome numbers vary even within a monophyletic lineage, which calls for further research (unpublished data). Currently, chloroplast genome sequences of related species are available (Kim et al. 2016a, c; Chen et al. 2020), which can be utilized to further elucidate the relationship among these species and their evolution that leads to the different characteristics.

## Conclusion

This review discussed the dynamic evolutionary history of *Panax* species that was revealed by whole genome sequence data of *P. ginseng* as well as comparative analysis with other related species. *P. ginseng* genome went through two rounds of whole genome duplication events throughout evolution that separately occurred after diverging from the closest family Apiaceae. Duplication events resulted in paralogous blocks within the genome that shows high sequence similarity. Sequence information such as repeat elements, paralogous genes, organellar genomes, and molecular cytogenetic tools were utilized to decipher the relationship among *Panax* species and detect clues for evolution and speciation. These results provide fundamental information for further ginseng research that can be used in various future studies such as molecular assisted breeding, and the understanding of evolutionary history of the *Panax* genus with other genera in the Araliaceae family.
